# Down-Regulation of *OsSPX1* Causes High Sensitivity to Cold and Oxidative Stresses in Rice Seedlings

**DOI:** 10.1371/journal.pone.0081849

**Published:** 2013-12-03

**Authors:** Chunchao Wang, Qiang Wei, Kang Zhang, Ling Wang, Fengxia Liu, Linna Zhao, Yuanjun Tan, Chao Di, Hong Yan, Jingjuan Yu, Chuanqing Sun, Wenqiong J. Chen, Wenying Xu, Zhen Su

**Affiliations:** 1 State Key Laboratory of Plant Physiology and Biochemistry, College of Biological Sciences, China Agricultural University, Beijing, China; 2 Department of Plant Genetic and Breeding and State Key Laboratory of Agrobiotechnology, China Agricultural University, Beijing, China; 3 State Key Laboratory for Agricultural Biotechnology, College of Biological Sciences, China Agricultural University, Beijing, China; 4 Biology Department, San Diego State University, San Diego, California, United States of America; Institute of Botany, Chinese Academy of Sciences, China

## Abstract

Rice SPX domain gene, *OsSPX1*, plays an important role in the phosphate (Pi) signaling network. Our previous work showed that constitutive overexpression of *OsSPX1* in tobacco and Arabidopsis plants improved cold tolerance while also decreasing total leaf Pi. In the present study, we generated rice antisense and sense transgenic lines of *OsSPX1* and found that down-regulation of *OsSPX1* caused high sensitivity to cold and oxidative stresses in rice seedlings. Compared to wild-type and *OsSPX1*-sense transgenic lines, more hydrogen peroxide accumulated in seedling leaves of *OsSPX1*-antisense transgenic lines for controls, cold and methyl viologen (MV) treatments. Glutathione as a ROS scavenger could protect the antisense transgenic lines from cold and MV stress. Rice whole genome GeneChip analysis showed that some oxidative-stress marker genes (e.g. glutathione S-transferase and P450s) and Pi-signaling pathway related genes (e.g. *OsPHO2*) were significantly down-regulated by the antisense of *OsSPX1*. The microarray results were validated by real-time RT-PCR. Our study indicated that *OsSPX1* may be involved in cross-talks between oxidative stress, cold stress and phosphate homeostasis in rice seedling leaves.

## Introduction

The SPX domain, a domain of 180 residues in length at the N-termini of the proteins, was defined after the SYG1/Pho81/XPR1 proteins. Many proteins possessing the SPX domain have been suggested to be involved in phosphate (Pi) signaling, for example, the N-terminus of yeast SYG1 binds to the G-protein beta subunit and inhibits mating pheromone signal transduction [1]. The putative Pi-level sensors, Pho81 and NUC-2, have a SPX domain in their N-termini and may be involved in regulation of Pi transport [2–4]. The human XPR1 functions as a Pi sensor and may be involved in G-protein associated signal transduction [5,6]. The SPX domain of the yeast low-affinity Pi transporter Pho90 was reported to regulate transport activity through physical interaction with Spl2 [7]. In plants, many SPX domain proteins were also identified as involved in the Pi-related signal transduction pathway and regulation pathways. Phosphorus (P) is well-known as a major macronutrient for plant growth and development. 

All SPX domain proteins in rice and Arabidopsis have been classified into four classes based on phylogenetic and domain analyses [8,9]. The four classes are differentiated by specific conserved domains: three rice and 11 Arabidopsis proteins in Class 1 (PHO1 and PHO1-like); six rice (OsSPX) and four Arabidopsis (AtSPX) proteins in Class 2; three Arabidopsis and six rice proteins (four rice genes) in Class 3; and two Arabidopsis and two rice proteins in Class 4. The PHO1 (At3g23430) and PHO1-like proteins were identified as involved in ion transport in Arabidopsis [10–13]. The Arabidopsis *pho1* mutant was characterized by severe deficiency in shoot Pi but normal root Pi content. *PHO1* is a gene specifically involved in the loading of Pi into the xylem in roots and is expressed in cells of the root vascular system [11]. Three members of the AtPHO1 family had possible interactions with signaling pathways involved in Pi deficiency and responses to auxin, cytokinin and abscisic acid [14]. The *PHO1* gene family was also identified in *Physcomitrella patens* and responded to Pi deficiency [12]. Arabidopsis AtSPX family genes, encoding another class of proteins with a SPX domain, have diverse functions in plant tolerance to phosphorus starvation [8]. *AtSPX1* showed 52-fold induction under Pi starvation [15]. The expression levels of *AtSPX1* and *AtSPX3* were induced by Pi starvation, *AtSPX2* was slightly induced and *AtSPX4* was suppressed. The *AtSPX* family may be part of the Pi-signaling pathways controlled by *PHR1* and *SIZ1* [8]. Three rice *OsSPX*s were identified as Pi-starvation response genes in rice seedlings [16]; *OsSPX1* is involved in Pi homeostasis through a negative feedback loop under Pi-limited conditions in rice [17]. *OsSPX1* was suggested to a regulator for the transcriptions of *OsSPX2*, 3 and 5; and *OsSPX3* negatively regulated the PSI (Pi-starvation induced) genes [18]. *OsSPX1* was reported to suppress the function of *OsPHR2* in the regulation of *OsPT2* expression and Pi homeostasis in rice shoots [19]. 

In general, plants regulate multiple metabolic processes to adapt to low Pi environments, such as altering lipid metabolism [20], increasing synthesis and activity of RNases and acid phosphatases, and changing the metabolic bypasses of glycolysis [21]. During Pi over-accumulation, some Pi-starvation related genes were reported to be involved in Pi toxicity: for example, rice plants over-expressing *OsPT8* showed Pi toxicity symptoms in leaves under high Pi supply [22]; mature leaves of *OsSPX1* RNAi plants showed necrotic spots under Pi-sufficient conditions [17]; *OsPHR2* over-expressing lines displayed chlorosis or necrosis on leaf margins at high Pi levels [23]; and *ltn1* (OsPHO2) mutant displayed leaf tip necrosis in mature leaves [24].

There is a close relationship between Pi-signaling and abiotic stresses, including cold stress, in plants. In Arabidopsis *pho1-2* and *pho2-1* mutants, Hurry et al. (2000) reported that low Pi played an important role in triggering cold acclimatization of leaf tissues [25]. Our previous study reported that constitutive overexpression of *OsSPX1* in tobacco and Arabidopsis plants improved cold tolerance while also decreasing total leaf Pi [9]. There have been some studies on the relationship between reactive oxygen species (ROS) signaling and cold stress [26–30]. In the present study, we generated rice antisense and sense transgenic lines of *OsSPX1* to study the role of *OsSPX1* involved in cold response and the possible relationship with oxidative stress. Furthermore, we conducted rice whole genome GeneChip analysis to elucidate the possible molecular mechanism underlying the down-regulation of *OsSPX1* causing high sensitivity to oxidative stress in rice seedling leaves.

## Results

### Generation of *OsSPX1* transgenic rice lines

**Figure 1:**
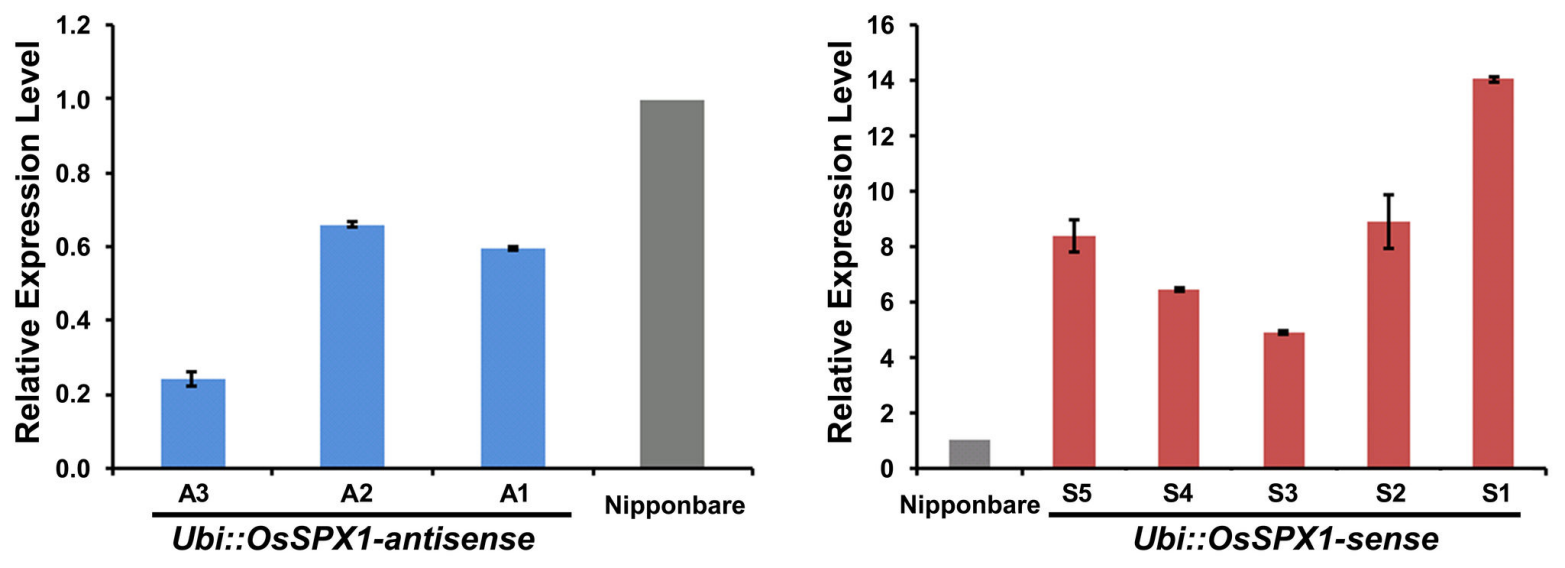
Real-time RT-PCR validation of transgenic rice lines. The expression levels of *OsSPX1* in WT (gray bars) and transgenic rice lines (blue bars for Ubi*::OsSPX1*-antisense lines and red bars for Ubi*::OsSPX1*-sense lines). Expression levels were normalized to 18S rRNA. The results are mean values ±SD of three replicates.

To characterize the gene function of *OsSPX1* in rice plants, we applied an antisense and sense transgenic approach. The cloned full-length cDNAs for OsSPX1 (adapted from our previous paper [9]) was used to generate transgenic rice lines with *Oryza sativa* ssp. *japonica* cv. Nipponbare as the wild-type (WT) background. The down-regulation of *OsSPX1* gene by antisense approach (Figure S1A) and the over-expressing *OsSPX1* gene by sense approach (Figure S1B) was under the control of the ubiquitin promoter. Several independent hygromycin-resistant transgenic lines were generated for *Ubi::OsSPX1*-antisense and -sense. Real-time RT-PCR was applied to determine the expression levels of *OsSPX1* in WT and transgenic rice lines, using different primers for *Ubi::OsSPX1*-antisense and -sense transgenic rice lines (primers are listed in Table S2). The T_3_ generation *Ubi::OsSPX1*-sense lines (lines S1-S5, on the right of Figure 1) accumulated a substantial amount of the transcript, whereas the level of transcript was very low in WT. For the T_3_ generation *Ubi::OsSPX1*-antisense lines (lines A1-A3, on the left of Figure 1), the expression level of *OsSPX1* was significantly lower than in WT plants (about 35% - 75% lower in *Ubi::OsSPX1*-antisense lines; by *t*-test, p< 0.05).

### Cold tolerance analysis of *OsSPX1* transgenic lines and WT rice seedlings

**Figure 2:**
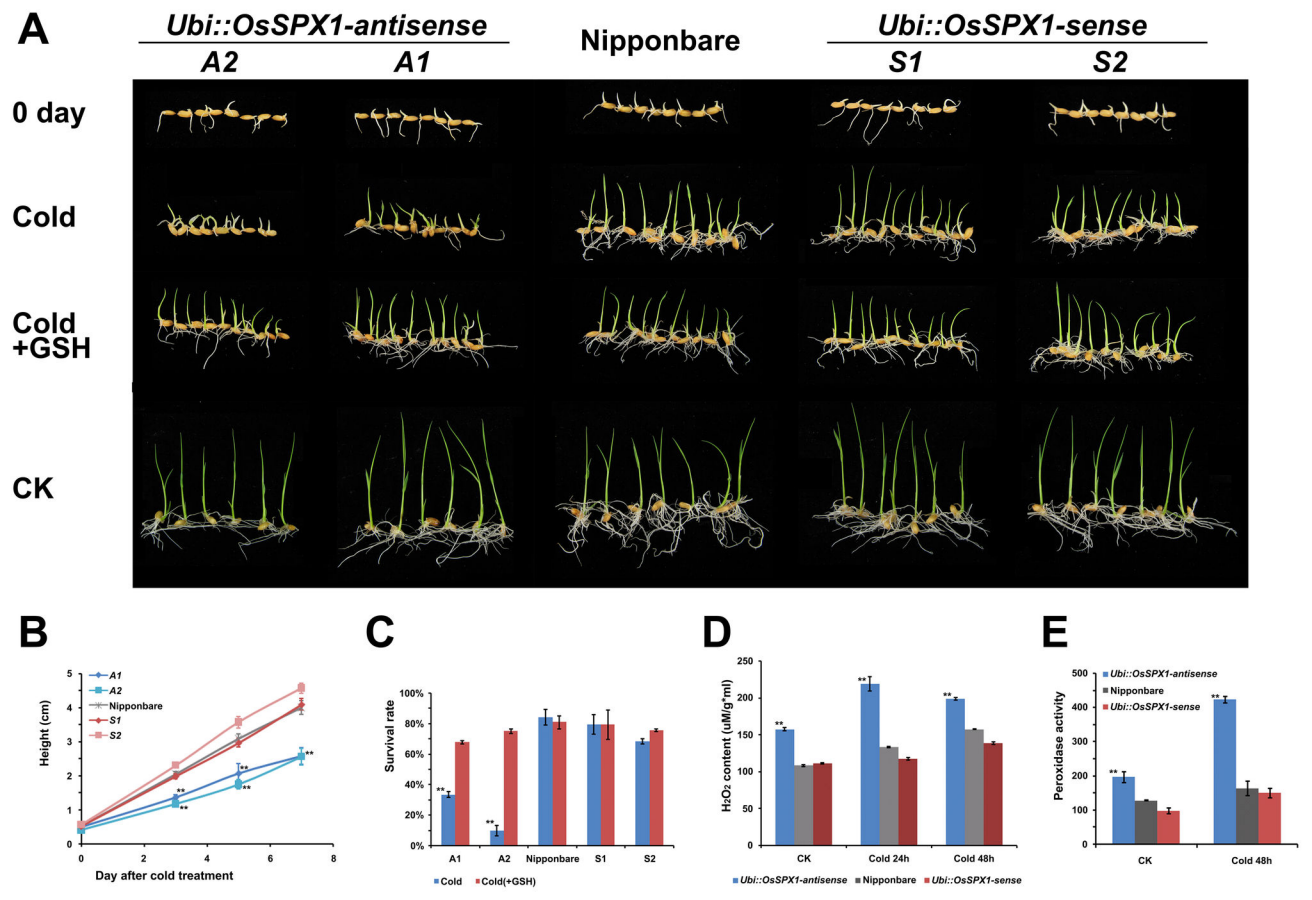
Characterization of the transgenic rice seedlings under cold treatment. A. 0 day - germinated seeds of WT and transgenic plants; CK - seedlings of the WT and transgenic plants grown for 6 d at room temperature (28°C); Cold - seedlings with 5 mm bud burst were treated at 4-5°C for 7 d, then recovered for 4 d at room temperature (28°C); Cold+GSH - the cold-treated seedlings were recovered under 10 mg/L GSH solution for 4 d at room temperature (28°C). A1 and A2 are Ubi*::OsSPX1*-antisense transgenic lines (on the left), S1 and S2 are Ubi*::OsSPX1*-sense lines (on the right). B. The height of the WT and transgenic plants over the recovery time after cold treatment (blue lines for Ubi*::OsSPX1*-antisense and red lines for Ubi*::OsSPX1*-sense). C. The survival rate of the WT and transgenic plants after cold treatment, with/without GSH. D. H_2_O_2_ content of the leaves of WT and transgenic rice plants under cold treatment (blue bars for Ubi*::OsSPX1*-antisense and red bars for Ubi*::OsSPX1*-sense). E. Peroxidase activity in the WT and transgenic rice plants under cold treatment (blue bars for Ubi*::OsSPX1*-antisense and red bars for Ubi*::OsSPX1*-sense). ** represent significant differences (p < 0.01) between transgenic and WT rice plants according to Student’s *t*-test.

The cold stress response of the *OsSPX1* transgenic rice (T_4_ generation plants of both antisense and sense transgenic lines) plants were tested together with WT at the seedling stage. The rice seedlings (WT and *Ubi::OsSPX1*-antisense and -sense lines) with 5-mm bud burst were treated at 4-5°C for 7 d and then recovered at room temperature (Figure 2A). There were no obvious differences between the WT and transgenic lines at room temperature for 6 d; however, after cold treatment the *Ubi::OsSPX1*-antisense plants grew slowly (or even died) and were significantly shorter than the WT and sense plants (Figure 2A and 2B). The height of seedlings was measured at 0, 3, 5 and 7 d after cold treatment. Starting from 3 d of recovery after cold treatment, the height of seedlings of two *Ubi::OsSPX1*-antisense transgenic lines (A1 and A2; the blue solid lines in Figure 2B) showed significant differences from WT (gray solid line in Figure 2B; *t*-test, p < 0.01) and sense lines (S1 and S2, the red solid lines in Figure 2B). Up to 7 d of recovery after cold treatment, the growth of several plants of the A1 and A2 lines ceased and some seedlings died (Figure 2C). The survival rates of A1 and A2 lines were significantly lower than WT plants (p < 0.01) and sense lines (S1 and S2). This phenotype indicated that the antisense plants were more cold-stress sensitive than WT and sense plants. 

In addition, we measured the hydrogen peroxide (H_2_O_2_) content in leaves of WT and transgenic rice plants (Figure 2D). Before cold treatment (i.e. at room temperature), the leaves of *Ubi::OsSPX1*-antisense plants accumulated much more H_2_O_2_ than did WT and sense plants. After 24 h of cold treatment, the leaf H_2_O_2_ content of the antisense plants increased about 40%, but that of WT plants increased about 20% and that of sense plants only increased about 5%. The leaf H_2_O_2_ content difference between the antisense transgenic lines and WT plants was significant (p < 0.01), as was the difference between sense line and WT plants. There was a similar trend after 48 h of cold treatment (Figure 2D). 

We also measured the peroxidase activity in the WT and transgenic plants (Figure 2E), and this showed a similar trend to leaf H_2_O_2_ content. Peroxidase activity was much higher in antisense plants than in WT and sense plants, both before and after cold treatment.

When adding the antioxidant, glutathione (GSH), into the cold treated rice plants, the cold-sensitive phenotype of the *Ubi::OsSPX1*-antisense transgenic plants was recovered (Figure 2A and C), including the growth rate and survival rate. 

### Differential response of *OsSPX1* transgenic and WT rice seedlings under methyl viologen (MV, or Paraquat) treatment

**Figure 3:**
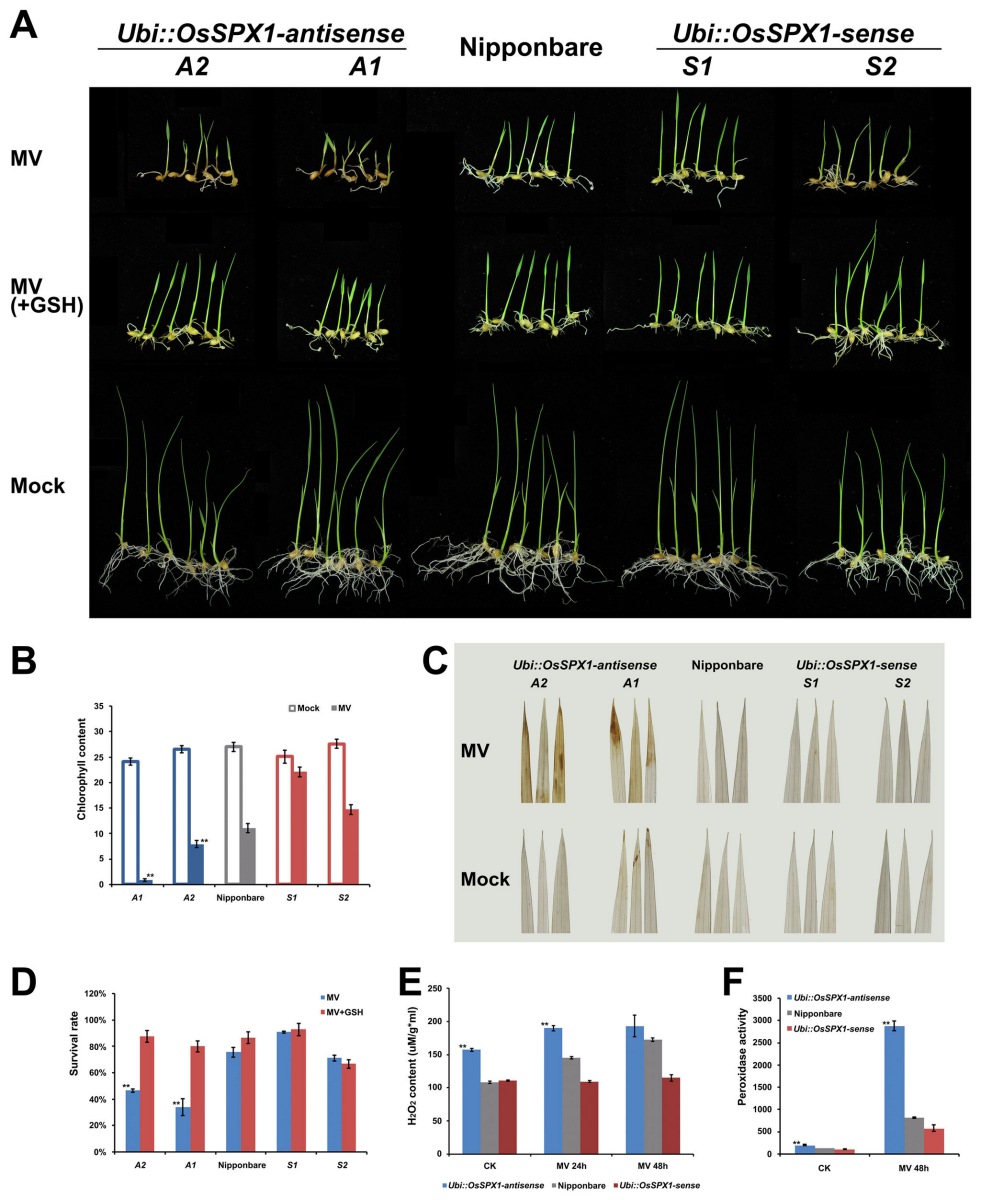
Differential response of WT and transgenic rice lines under MV treatment. A. Four-day-old seedlings treated with 10 μM MV (MV) and 10 μM MV plus 10 mg/L GSH (MV+GSH) for 3 d, water as mock. A1 and A2 are Ubi*::OsSPX1*-antisense transgenic lines (on the left), S1 and S2 are Ubi*::OsSPX1*-sense lines (on the right). B. The chlorophyll content of seedlings under MV treatment (blue bars for Ubi*::OsSPX1*-antisense and red bars for Ubi*::OsSPX1*-sense). C. DAB staining of the seedling leaves treated with 10 μM MV, water as mock. A1 and A2 are Ubi*::OsSPX1*-antisense transgenic lines (on the left), S1 and S2 are Ubi*::OsSPX1*-sense lines (on the right). D. The survival rate of WT and transgenic plants treated with 10 μM MV, with/without GSH. E. H_2_O_2_ content of the leaves of WT and transgenic plants under MV treatment (blue bars for Ubi*::OsSPX1*-antisense and red bars for Ubi*::OsSPX1*-sense). F. Peroxidase activity in WT and transgenic rice plants under MV treatment (blue bars for Ubi*::OsSPX1*-antisense and red bars for Ubi*::OsSPX1*-sense). ** represent significant differences (p < 0.01) between transgenic and WT rice plants according to Student’s *t*-test.

The significant difference in H_2_O_2_ content and peroxidase activity between WT and transgenic rice plants under normal condition and cold treatment indicated that the differential cold-stress responses of *OsSPX1* transgenic lines may be related to oxidant response activity. We performed a MV treatment experiment to test our hypothesis. Four-day-old seedlings of WT, *Ubi::OsSPX1*-antisense and -sense transgenic lines were transferred to 10 μM MV solution, with water as mock. After 3 d of growth, the differences in leaf phenotype became significant between antisense lines (A1 and A2) and the WT and sense lines (S1 and S2) (Figure 3A). In the mock condition, WT and transgenic seedlings grew normally, and all leaves were green. Under MV treatment, both WT and transgenic seedlings were dwarfed. However, there was significant phenotype divergence: the leaves of A1 and A2 seedlings became scorched and yellow with little green, their seedling leaves presented lesions and then died; in contrast, S1 and S2 plants grew better, and the seedling leaves were still green after 3 d of MV treatment (Figure 3A). The detected leaf chlorophyll contents also confirmed the leaf phenotype (Figure 3B). Leaf chlorophyll contents were similar in WT and transgenic plants during mock condition; however, under MV solution, the leaf chlorophyll contents of A1 and A2 lines were significantly lower than that of WT (p < 0.01) and lines S1 and S2. The survival rate of A1 and A2 lines under MV treatment were also significantly lower than that of WT (p < 0.01) and sense lines (Figure 3D).

In addition, we conducted 3,3’-diaminobenzidine (DAB) staining to detect the accumulation of H_2_O_2_ in rice leaves (Figure 3C). Under MV treatment, the leaves of *Ubi::OsSPX1*-antisense transgenic plants were significantly stained dark brown while WT and sense leaves were lighter or not stained. Moreover, the direct measurement of H_2_O_2_ also indicated that the leaves of antisense plants accumulated much more H_2_O_2_ than did WT and sense plants with and without MV treatment (p < 0.01, Figure 3E). The peroxidase activity of rice seedlings showed a similar trend to the H_2_O_2_ content (Figure 3F). Overall, the WT and sense seedlings showed strong oxidative stress resistance compared to antisense seedlings in the MV experiment. As for cold treatment, when GSH solution was added to the MV, the sensitive phenotype of *Ubi::OsSPX1*-antisense transgenic plants under MV treatment was recovered (Figure 3A and D).

In general, H_2_O_2_ is one of the ROS that leads to programmed cell death (PCD), but it also acts as a signal molecule in various biological processes including cold response. The high level of H_2_O_2_ in *Ubi::OsSPX1*-antisense transgenic plants led us to hypothesize that down-regulation of *OsSPX1* resulted in a reduced ability to eliminate of ROS. Thus we designed gene expression experiments to determine molecular relationships between cold stress and ROS controlled by *OsSPX1*. 

### Transcriptome analysis of *Ubi*::*OsSPX1*-antisense transgenic and WT rice seedlings

GeneChip^®^ microarray transcriptome experiments were performed with seedlings collected from the WT rice and two *Ubi::OsSPX1*-antisense transgenic lines (A1 and A2) under cold treatment and normal conditions. The change in expression level for each probe set between antisense lines and WT was conducted by MAS5 algorithm using the GCOS baseline tool. Then one-sample *t*-test were applied to the log_2_ratios to identify differentially expressed probe sets. Using the cut-off (p ≤ 0.05 and log_2_ratio ≥ 0.6), we identified a total of 1266 probe sets, including 748 up-regulated and 518 down-regulated in *Ubi::OsSPX1*-antisense transgenic lines (detailed information on these probe sets is listed in Table S1). 

We further applied gene ontology (GO) enrichment analysis (by EasyGO tool [31]) on these differentially expressed probe sets and determined the significant GO terms (FDR p ≤ 0.05 as cut-off; Table 1). For the 518 probe sets down-regulated in *Ubi::OsSPX1*-antisense transgenic lines, several GO terms including response to toxin, response to chemical stimulus, potassium ion symporter activity, and glutathione transferase activity were significantly enriched. These enriched GO categories indicated that the differentially expressed probe sets may be related to oxidative process. For the 748 probe sets up-regulated in antisense lines, the enriched GO terms included sugar mediated signaling, photosynthesis, NADH dehydrogenase activity, and hormone-related GO terms (GA and ABA). 

**Table 1:** GO analysis of differentially expressed probe sets between *Ubi::OsSPX1*-antisense transgenic lines and WT (Nipponbare).

**GO term**	**GO name**	**Qnum**	**B/Rnum**	**FDR p-value**
**518 probe sets down-regulated in *Ubi::OsSPX1*-antisense transgenic lines**				
**Biological Process**		**265**	**21427**	
GO:0009407	toxin catabolic process	8	89	5.08E-06
GO:0009404	toxin metabolic process	8	113	4.01E-04
GO:0009626	plant-type hypersensitive response	11	218	1.32E-03
GO:0009636	response to toxin	8	130	1.83E-03
GO:0009812	flavonoid metabolic process	6	105	3.26E-02
GO:0009813	flavonoid biosynthetic process	6	105	3.26E-02
GO:0042221	response to chemical stimulus	34	1509	4.02E-02
**Molecular Function**		**320**	**27148**	
GO:0008393	fatty acid (omega-1)-hydroxylase activity	3	5	2.82E-12
GO:0009674	potassium:sodium symporter activity	3	10	8.22E-07
GO:0022820	potassium ion symporter activity	3	10	8.22E-07
GO:0004364	glutathione transferase activity	9	179	7.28E-03
GO:0008493	tetracycline transporter activity	3	21	8.84E-03
**Cellular Component**		**338**	**29111**	
GO:0012505	endomembrane system	90	5277	8.84E-03
**748 probe sets up-regulated in *Ubi::OsSPX1*-antisense transgenic lines**				
**Biological Process**		**370**	**21427**	
GO:0006270	DNA replication initiation	8	19	1.49E-22
GO:0009687	abscisic acid metabolic process	6	21	3.16E-11
GO:0009688	abscisic acid biosynthetic process	6	21	3.16E-11
GO:0015979	photosynthesis	24	387	9.08E-08
GO:0009405	pathogenesis	13	157	4.20E-06
GO:0006306	DNA methylation	4	20	2.83E-04
GO:0009686	gibberellin biosynthetic process	5	33	3.03E-04
GO:0007018	microtubule-based movement	8	85	4.26E-04
GO:0009685	gibberellin metabolic process	5	44	9.08E-03
GO:0010182	sugar mediated signaling	4	32	2.92E-02
GO:0009814	defense response, incompatible interaction	11	202	3.55E-02
GO:0015712	hexose phosphate transport	4	33	3.55E-02
**Molecular Function**		**439**	**27148**	
GO:0001882	nucleoside binding	3	5	4.95E-08
GO:0003954	NADH dehydrogenase activity	33	656	5.14E-08
GO:0003886	DNA (cytosine-5-)-methyltransferase activity	3	7	4.03E-06
GO:0008137	NADH dehydrogenase (ubiquinone) activity	7	55	1.73E-05
GO:0016651	oxidoreductase activity, acting on NADH or NADPH	36	898	2.82E-05
GO:0051539	4 iron, 4 sulfur cluster binding	3	11	6.24E-04
GO:0003777	microtubule motor activity	9	127	6.61E-03
GO:0003989	acetyl-CoA carboxylase activity	14	275	1.09E-02
GO:0008094	DNA-dependent ATPase activity	8	109	1.13E-02
GO:0008254	3'-nucleotidase activity	3	18	4.30E-02
GO:0019133	choline monooxygenase activity	3	18	4.30E-02
**Cellular Component**		**491**	**29111**	
GO:0009512	cytochrome b6f complex	13	101	2.74E-12
GO:0043601	nuclear replisome	3	5	9.53E-09
GO:0009579	thylakoid	29	549	7.03E-08
GO:0009523	photosystem II	21	367	2.22E-06
GO:0009522	photosystem I	12	167	5.86E-05
GO:0044436	thylakoid part	16	306	7.08E-04
GO:0009539	photosystem II reaction center	6	66	4.93E-03
GO:0005875	microtubule associated complex	8	131	3.57E-02
GO:0009941	chloroplast envelope	13	295	4.91E-02
GO:0043245	extraorganismal space	10	197	4.91E-02

**Figure 4:**
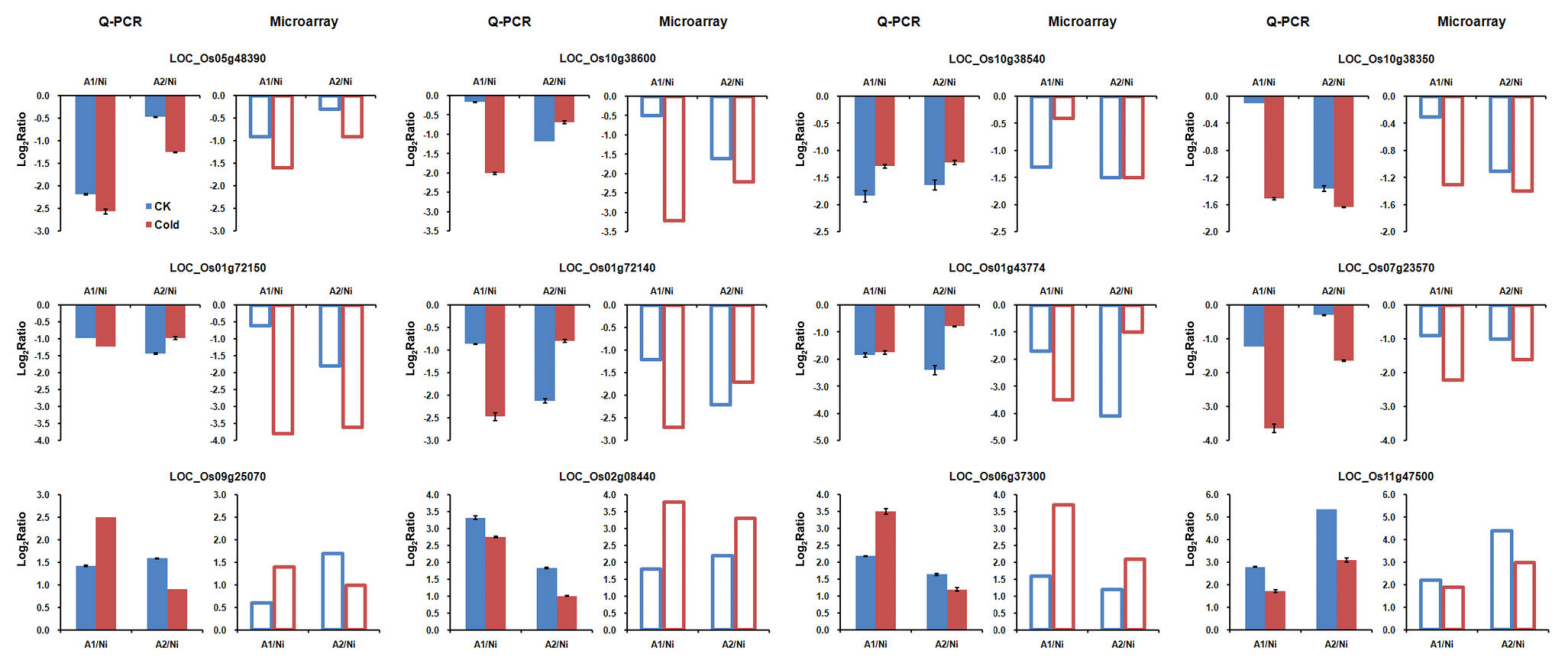
Real-time RT-PCR validation for selected probe sets. Twelve genes were selected for real-time RT-PCR to validate the expression patterns of Ubi*::OsSPX1*-antisense transgenic lines (A1 and A2) and WT rice (Ni) seedlings under control condition and cold treatment. These genes are: LOC_Os05g48390 - OsPHO2, ubiquitin conjugating enzyme; LOC_Os10g38600 - glutathione S-transferase GSTU6; LOC_Os10g38540 - glutathione S-transferase GSTU6; LOC_Os10g38350 - glutathione S-transferase GSTU6; LOC_Os01g72150 - glutathione S-transferase; LOC_Os01g72140 - glutathione S-transferase; LOC_Os01g43774 - cytochrome P450 CYP72A23; LOC_Os07g23570 - cytochrome P450 CYP709C9; LOC_Os09g25070 - OsWRKY62; LOC_Os02g08440 - OsWRKY71; LOC_Os06g37300 - ent-kaurene oxidase; LOC_Os11g47500 - xylanase inhibitor protein 1 precursor. The error bars of real-time RT-PCR results represent the standard error from three independent biological replicates.

Furthermore, we selected several genes for real-time RT-PCR confirmation, such as OsPHO2, GSTs, P450s and WRKYs. Additional biological samples were collected for real-time RT-PCR validation. For each selected gene, the log_2_ratio between two *Ubi::OsSPX1*-antisense transgenic lines (A1 and A2) and the WT under control and cold conditions are shown in Figure 4 - the corresponding log_2_ratio of microarray results is also shown. One-by-one comparison validated the majority of microarray results with real-time RT-PCR results in these selected genes, most of them showed similar patterns.

From these GO terms and some important gene families, some key genes were highlighted. There were nine glutathione S-transferase (GST) genes in the differentially expressed probe sets, but only one was up-regulated in *Ubi::OsSPX1-antisense* transgenic lines (Table S1). Among the other eight down-regulated GSTs, six were up-regulated by MV treatment (Table 2); moreover, four of the six GSTs were located on the rice chromosome 10, including the GST gene cluster: LOC_Os10g38340, LOC_Os10g38350 and LOC_Os10g38360. Of cytochrome P450 genes, several family members were up-regulated in *Ubi::OsSPX1*-antisense transgenic lines, such as CYP71 and CYP94 families; while several family members were down-regulated in antisense lines, such as CYP72A, CYP709 and CYP86A subfamilies (Table S1). Notably two P450 genes, CYP72A23 and CYP709C9, were down-regulated in antisense lines and up-regulated by MV treatment (Table 2). There were some other family genes shown preference in the differentially expressed probe sets, for examples, many peroxidases (including OsAPx3) and UDP-glucoronosyl transferases were down-regulated in antisense lines, and several WRKY family members were up-regulated in antisense lines. 

**Table 2:** Differentially expressed probe sets between *Ubi::OsSPX1*-antisense transgenic lines and WT (Nipponbare) related to MV treatment.

		***Ubi::OsSPX1*-antisense vs Ni**		**MV vs CK (Ni**)^(a)^			
**Probe Set ID**		**Log_2_Ratio**	**p-value**	**Log_2_Ratio**	**p-value**	**Locus ID**	**Annotation**
Os.40000.1.S1_at		-1.63		4.07E-02	1.58	1.28E-05	LOC_Os10g38600	glutathione S-transferase GSTU6
Os.40000.1.S1_x_at		-1.88		4.51E-02	1.70	2.58E-05	LOC_Os10g38600	glutathione S-transferase GSTU6
Os.13015.1.S1_at		-1.25		4.80E-02	2.92	1.31E-03	LOC_Os10g38360	glutathione S-transferase GSTU6
Os.46635.1.S1_x_at		-1.03		2.61E-02	2.41	2.66E-04	LOC_Os10g38350	glutathione S-transferase GSTU6
Os.9013.1.S1_at		-2.00		3.77E-02	4.22	7.38E-04	LOC_Os10g38340	glutathione S-transferase GSTU6
OsAffx.24011.1.S1_at		-2.45		4.90E-02	4.31	4.60E-05	LOC_Os01g72150	glutathione S-transferase
Os.22957.1.S1_at		-1.95		9.09E-03	3.36	3.09E-06	LOC_Os01g72140	glutathione S-transferase
Os.9017.1.S1_x_at		-1.43		1.79E-02	3.79	4.10E-05	LOC_Os07g23570	cytochrome P450 CYP709C9
Os.41199.1.A1_at		-2.58		3.90E-02	6.35	5.21E-04	LOC_Os01g43774	cytochrome P450 CYP72A23
Os.56112.1.A1_at		-2.08		3.12E-02	1.93	3.94E-02	LOC_Os01g43774	cytochrome P450 CYP72A23

(a) The methyl viologen (MV) transcriptome data from [32]

We further used real-time RT-PCR to investigate the expression levels of GST and P450 genes affected by *OsSPX1* under oxidative stress. We detected the expression pattern of these genes in antisense line (A1), sense line (S1), and WT plants under MV treatment (Table 3). All these genes - including GST genes in chromosome 10 GST cluster, CYP72A23 and CYP709C9 - were significantly down-regulated in *Ubi::OsSPX1*-antisense transgenic line compared to WT and *Ubi::OsSPX1*-sense transgenic line (*t*-test, p < 0.05).

**Table 3:** Real-time RT-PCR for selected genes in transgenic lines and Nipponbare under MV treatment.

	**A1 vs Ni** ^(a)^		**A1 vs S1** ^(b)^		
**Locus ID**	**Log_2_Ratio**	**p-value**	**Log_2_Ratio**	**p-value**	**Annotation**
LOC_Os06g40120	-2.40	1.08E-02	-6.83	1.30E-06	OsSPX1
LOC_Os10g38600	-0.75	2.60E-04	-0.74	9.93E-03	glutathione S-transferase GSTU6
LOC_Os10g38540	-1.72	3.43E-03	-2.39	9.26E-04	glutathione S-transferase GSTU6
LOC_Os10g38360	-0.86	4.30E-03	-0.40	1.77E-02	glutathione S-transferase GSTU6
LOC_Os10g38350	-1.15	2.09E-03	-0.78	4.02E-02	glutathione S-transferase GSTU6
LOC_Os10g38340	-1.99	1.45E-04	-1.76	9.54E-03	glutathione S-transferase GSTU6
LOC_Os01g72150	-1.22	5.83E-03	-1.77	1.11E-03	glutathione S-transferase
LOC_Os01g72140	-1.01	3.18E-02	-1.47	1.40E-02	glutathione S-transferase
LOC_Os07g23570	-1.63	8.26E-05	-0.84	5.08E-02	cytochrome P450 CYP709C9
LOC_Os01g43774	-0.87	8.52E-04	-0.38	1.46E-02	cytochrome P450 CYP72A23

(a) A1 is *Ubi::OsSPX1*-antisense transgenic line and Ni is WT rice

(b) S1 is *Ubi::OsSPX1*-sense transgenic line

## Discussion

*OsSPX1* plays an important role in Pi homeostasis by controlling several biological processes [8,9,17,19]. It was reported that excessive Pi accumulation in seedling leaves of *OsSPX1*-RNAi rice plants caused toxicity [17]. Our previous work reported that increased expression of *OsSPX1* enhances cold/subfreezing tolerance in tobacco and *Arabidopsis thaliana* [9]. In the present study, we further elucidated the role of *OsSPX1* in cold response and the possible relationship with oxidative stress in rice seedlings. We applied an antisense and sense transgenic approach, and generated several individual *Ubi::OsSPX1*-antisense and -sense transgenic lines (Figure 1). The cold tolerance experiment on rice seedlings showed that *Ubi::OsSPX1*-antisense lines were significantly sensitive to cold stress (Figure 2A, 2B and 2C). We also detected more H_2_O_2_ accumulation in the seedling leaves of *Ubi::OsSPX1*-antisense lines, both in control and cold conditions (Figure 2D). H_2_O_2_, as a key ROS molecule, is induced by various biotic or abiotic stresses (including cold stress) and damages cellular macromolecules such as lipids, enzymes and DNA. The oxidative resistant ability of plants may be correlated with their resistance to cold/chilling stress. We further used MV, a widely used oxidant forming the toxic superoxide radical [32–34], to test the oxidative response of *Ubi::OsSPX1*-antisense and -sense transgenic and WT rice plants (Figure 3). There was a positive correlation between the cold and oxidation response in these rice plants, the antisense lines were highly sensitive to cold and oxidative stresses compared with sense lines and WT plants. H_2_O_2_ accumulation was significantly higher in seedling leaves of antisense than in sense lines and WT plants, both in control and MV treatment (Figure 3E). 

We conducted the transcriptome analysis with rice whole genome GeneChip to elucidate the possible molecular mechanism underlying the down-regulation of *OsSPX1* that caused high sensitivity to cold and oxidative stress in rice seedlings. We identified 1266 differentially expressed probe sets (including 748 up-regulated and 518 down-regulated in *Ubi::OsSPX1*-antisense lines). Transcriptome data analysis and real-time RT-PCR confirmed that several Pi-signaling pathway related genes were affected by down-regulation of *OsSPX1*, for example, *OsPHO2* (LOC_Os05g48390) was down-regulated in *Ubi::OsSPX1*-antisense transgenic lines (Figure 4 and Table S1). The mutant of *OsPHO2*, also named *ltn1*, displayed leaf tip necrosis predominantly and increased the uptake and translocation of Pi [24]. Arabidopsis *pho2* mutants, with increased shoot Pi, were shown to be more sensitive to freezing than WT after cold acclimatization [25]. Besides *OsPHO2*, there were several phospholipid biosynthesis-related genes also down-regulated in *Ubi::OsSPX1*-antisense transgenic lines (shown in Table S1), such as glycerol-3-phosphate acyltransferases, phospholipase D and patatin T5 precursors. Two rice RNase T2 [35] genes (LOC_Os09g36680 and LOC_Os09g36700) were up-regulated in *Ubi::OsSPX1*-antisense transgenic lines. Their orthologs, RNS2 in Arabidopsis and AhSL28 in Antirrhinum, were all induced during leaf senescence and Pi starvation [36,37]. The RNase activities were also activated in *ltn1* (*ospho2*) mutant under Pi-sufficient conditions [24].

GO analysis showed that several GO terms related to oxidative process were enriched in the differentially expressed probe sets. For examples, GSTs (EC 2.5.1.18) were included in GO terms ‘response to toxin’ and ‘glutathione transferase activity’, and enriched in the 518 probe sets down-regulated in the *Ubi::OsSPX1*-antisense transgenic lines. The substrate of GSTs, GSH is an antioxidant [38] and helps to clear the harmful components (including H_2_O_2_) in the cell [39]. We highlight the GST genes, especially for the hotspot GST gene cluster region in rice chromosome 10 (Table 2) - most of these genes were down-regulated in the *Ubi::OsSPX1*-antisense transgenic lines and induced by MV treatment in seedlings [32]. Moreover, these genes were also down-regulated in the antisense line under MV treatment (Table 3). Interestingly, GSH recovered the sensitive phenotype of antisense plants under cold and MV treatment (Figures 2A and 3A) and increased their survival rate (Figures 2C and 3D). This indicated that the lower expression level of GST genes in the *Ubi::OsSPX1*-antisense transgenic plants affected the GSH level and ROS homeostasis, and caused the antisense lines to be highly sensitive to cold and oxidative stresses.

In additional, several P450 genes (e.g. LOC_Os07g23570 - CYP709C9) were also down-regulated in *Ubi::OsSPX1*-antisense transgenic lines in control and cold and MV treatments, and induced by MV treatment (Tables 2 and 3). Rice *CYP709C9* was considered a possible ortholog of wheat P450 genes *CYP709C1* and *CYP709C3v2* [32], both of which were related to defence response [40,41]. These GSTs and P450s were reported as potential marker genes for rice oxidative stress tolerance [32]. In *Ubi::OsSPX1*-antisense transgenic lines, all these genes were down-regulated compared to WT either in normal condition or under cold treatment. This suggested that the high sensitivity to cold and oxidative stresses from down-regulation of *OsSPX1* may be caused by the influence on the expression level of these GSTs and P450s. Furthermore, these genes were also down-regulated in *Ubi::OsSPX1*-antisense transgenic lines compared to sense lines and WT under MV treatment (Table 3), suggesting that *OsSPX1* may be involved in broad biological processes related to oxidative stress. 

For two other detoxification enzymes, peroxidases and UDP-glucoronosyl transferase, the majority of differentially expressed genes were down-regulated in *Ubi::OsSPX1*-antisense transgenic lines (Table S1). Peroxidases are one group of H_2_O_2_-scavenging enzymes [42]; and both gene expression and activity of cytosolic ascorbate peroxidase (APX) strongly decreased during the activation of PCD [43]. We also noted that *OsAPx3* (LOC_Os04g14680, peroxisomal ascorbate peroxidase) and L-ascorbate oxidase (LOC_Os11g42220) were down-regulated in *Ubi::OsSPX1*-antisense lines, which may be correlated with the higher H_2_O_2_ level in these transgenic lines. Two NADP-dependent oxidoreductases (LOC_Os11g14910 and LOC_Os12g12470) were also down-regulated in *Ubi::OsSPX1*-antisense transgenic lines, indicating a weak antioxidant capacity. There are some reports concerning the relationship between UGT (UDP-glucoronosyl transferase) genes and oxidative stresses [33,44].

Of the up-regulated genes in *Ubi::OsSPX1*-antisense transgenic lines, several GO terms, such as ‘sugar mediated signalling’ and ‘hexose phosphate transport’, were enriched - this may be related to the crosstalk among the cold acclimatization, Pi starvation and sugar signalling pathway [45]. Some GO terms related to abscisic acid, gibberellin and pathogenesis were also enriched. Several CYP94 members (subfamily of P450 genes; Table S1) were also up-regulated in antisense lines, which are related to the JA signalling pathway [46,47]. JA antagonizes the ABA pathway in response to multiple abiotic and biotic stresses [48].

We identified about 100 cold-stress related genes through gene annotation, GO annotation and from the literature. We studied the expression pattern of these genes between *Ubi::OsSPX1*-antisense transgenic lines and WT plants under control and cold treatment (Table S3). Only one gene (OsICE1, LOC_Os11g32100) belonged to the 1266 differentially expressed probe sets, and other 17 genes showed differential expression under cold treatment, including several *OsDREB*s which were up-regulated in antisense lines. During the experiment, we also studied the effects of *OsSPX1* in other abiotic stresses, such as salt and osmotic. However, our preliminary results showed no significantly different phenotypes among *Ubi::OsSPX1*-antisense and -sense lines and WT seedlings (data not shown). It seems that *OsSPX1* may play a specific role in cross-talks between oxidative stress and cold stress. 

In summary, we generated rice antisense and sense transgenic lines for *OsSPX1* and discovered that down-regulation of *OsSPX1* caused more H_2_O_2_ accumulation in leaves and high sensitivity to cold and oxidative stresses in rice seedlings. Rice whole genome GeneChip and GO enrichment analyses indicated that the GO terms ‘response to toxin’ and oxidative process genes such as GST genes were significantly enriched in the probe sets down-regulated by the antisense of *OsSPX1*. This may provide a clue concerning the possible molecular mechanism explaining why *OsSPX1* down-regulation caused high sensitivity to cold and oxidative stress in rice seedling leaves. GSH could protect the *Ubi::OsSPX1*-antisense transgenic lines from cold and MV treatments. There may be some signaling links between cold hypersensitivity and toxicity induced by Pi accumulation in plant leaves, both of which may be related to H_2_O_2_ abundance causing oxidative stress. Our study may greatly benefit in plant protection from Pi toxicity and abiotic stresses, and improving growth and crop yield.

## Materials and Methods

### Plant materials

Seeds of rice (Nipponbare as WT, and *Ubi::OsSPX1*-antisense and *Ubi::OsSPX1*-sense transgenic lines) were surface-sterilized in 5% (w/v) sodium hypochlorite for 20 min and then washed in distilled water three or four times, then germinated in water for 2 d at room temperature and 1 d at 37°C. The seedlings were transferred to water-saturated Whatman filter paper and grown in a greenhouse (28°C / 25°C and 12 /12 h of day/night, and 83% relative humidity).

#### For RNA isolation

fresh seedlings were harvested after 24h of cold (4°C) treatment, or 10μM MV treatment; control plants under normal condition were also harvested at the same time. 

#### For phenotype evaluation

to identify the phenotype of rice (Nipponbare as WT, and *Ubi::OsSPX1*-antisense and -sense transgenic lines) under cold treatment, the germinated seeds with 5-mm bud burst were transferred to 4-5°C for 7 d, then the seedlings were recovered at room temperature in a greenhouse. For identification of the rice phenotype under MV treatment, we transferred the four-day-old seedlings of rice lines to mock (water) and 10 μM MV solution, and the phenotype of each line was investigated and recorded. To protect from cold and MV treatment, 10mg/L GSH was added.

### Construction of transgenic rice lines

 We mainly followed a previous method [9] to clone the sense and antisense of the full-length cDNA of *OsSPX1* into the binary vector pCOU controlled by the ubiquitin promoter. The recombinant plasmids were then introduced into *Agrobacterium tumefaciens* EHA105 strain following the freeze-thaw method. Transgenic rice lines were obtained by using calli derived from mature embryos of Nipponbare [49]. The concentration of the selected antibiotic, hygromycin B, was 50 mg/L.

### RNA isolation and real-time RT-PCR

All seedling samples from *Ubi::OsSPX1*-antisense transgenic lines and WT under cold treatment and normal condition were homogenized in liquid nitrogen before isolation of the RNA. Total RNA was isolated using TRIZOL^®^ reagent (Invitrogen, CA, USA) and purified using Qiagen RNeasy columns (Qiagen, Hilden, Germany). Reverse transcription was performed using Moloney murine leukemia virus (M-MLV; Invitrogen). We heated 10-µl samples containing 2 µg of total RNA, and 20 pmol of random hexamers (Invitrogen) at 70°C for 2 min to denature the RNA, and then chilled the samples on ice for 2 min. We added reaction buffer and M-MLV to a total volume of 20 µL containing 500 µM dNTPs, 50 mM Tris–HCl (pH 8.3), 75 mM KCl, 3 mM MgCl_2_, 5mM dithiothreitol, 200 units of M-MLV and 20 pmol random hexamers. The samples were then heated at 42°C for 1.5 h. The cDNA samples were diluted to 8 ng/µL for real-time RT-PCR analysis. 

For real-time RT-PCR, triplicate quantitative assays were performed on 1µL of each cDNA dilution using the SYBR Green Master Mix (Applied Biosystems, PN 4309155) with an ABI 7900 sequence detection system according to the manufacturer’s protocol (Applied Biosystems). The gene-specific primers were designed by using PRIMER3 (http://frodo.wi.mit.edu/primer3/input.htm). The amplification of 18S rRNA was used as an internal control to normalize all data (forward primer, 5’-CGGCTACCACATCCAAGGAA-3’; reverse primer, 5’- TGTCACTACCTCCCCGTGTCA-3’). Gene-specific primers are listed in Table S2. The relative quantification method (ΔΔCT) was used to evaluate quantitative variation between replicates examined.

### Affymetrix GeneChip Analysis

For each sample, 8 μg of total RNA was used for making biotin-labeled cRNA targets. All processes concerning cDNA and cRNA synthesis, cRNA fragmentation, hybridization, washing and staining, and scanning, followed the GeneChip Standard Protocol (Eukaryotic Target Preparation). In this experiment, Poly-A RNA Control Kit and the One-Cycle cDNA Synthesis kit were applied. Affymetrix GCOS software was used for data normalization and comparative analysis. The change of expression level for each probe set between each *Ubi::OsSPX1*-antisense transgenic line and WT rice sample was calculated by MAS5 algorithm through GCOS baseline tool. One-sample *t*-test was applied onto the log_2_ratio values to identify the differentially expressed probe sets.

In order to map the probe set ID to the locus ID in the rice genome, the consensus sequence of each probe set was compared by BLAST (Basic Local Alignment and Search Tool) against the TIGR Rice Genome version 5. The cut-off e-value was set as 1e^-20^. EasyGO [31] tool was used for gene functional categorization.

### Chlorophyll content and H_2_O_2_/peroxidase measurements

Chlorophyll relative content was measured using a SPAD-502 Chlorophyll Meter. Each rice leaf was measured 3-4 times, and about 20 leaves were detected and calculated.

Five days after 10 μM MV treatment, staining with DAB (a H_2_O_2_ staining agent) was performed as described in the references 50,51. Seedlings were stained with DAB for 12 h in the darkness, then destained with acetic acid/glycerol/ethanol (1:1:3) at 100°C for 15 min. Representative leaves were viewed under a stereo fluorescence microscope.

Using the Amplex Red hydrogen peroxide/peroxidase assay kit (Molecular Probes), we followed the manufacturer’s instructions to measure the quantity of H_2_O_2_ production in rice leave samples and the peroxidase activity in rice samples.

## Supporting Information

Figure S1 **Schematic diagram of the draft structure of binary vector structures used for rice transformation.** Construction scheme of plasmids with *OsSPX1* in sense and antisense orientation.
**A**: The binary vector pCOU *OsSPX1*-antisense was used for transgenic rice transformation.
**B**: The binary vector pCOU *OsSPX1*-sense was used for transgenic rice transformation, which was adapted from our previous work [9].
LB and RB correspond to the T-DNA left and right borders.
(JPG)

Table S1 **The 1266 probe sets showing differential expression between *Ubi::OsSPX1*-antisense transgenic lines and WT seedlings.** Including the raw intensity, log2ratio, p-value, and additional annotation of each probe set.
(XLS)

Table S2 **Primer list of probe sets for real-time RT-PCR.**
(DOC)

Table S3 **Cold-stress related probe sets showing differential expression between *Ubi::OsSPX1*-antisense transgenic lines and WT seedlings under cold treatment.** Including the raw intensity, log2ratio, p-value, and additional annotation of each probe set.
(XLS)
