# Supplementary figures and images for: Down-Regulation of OsSPX1 Causes High Sensitivity to Cold and Oxidative Stresses in Rice Seedlings
Source: PLoS One. 2013 Dec 3;8(12):e81849. doi: 10.1371/journal.pone.0081849 (PMC3849359; doi:10.1371/journal.pone.0081849)

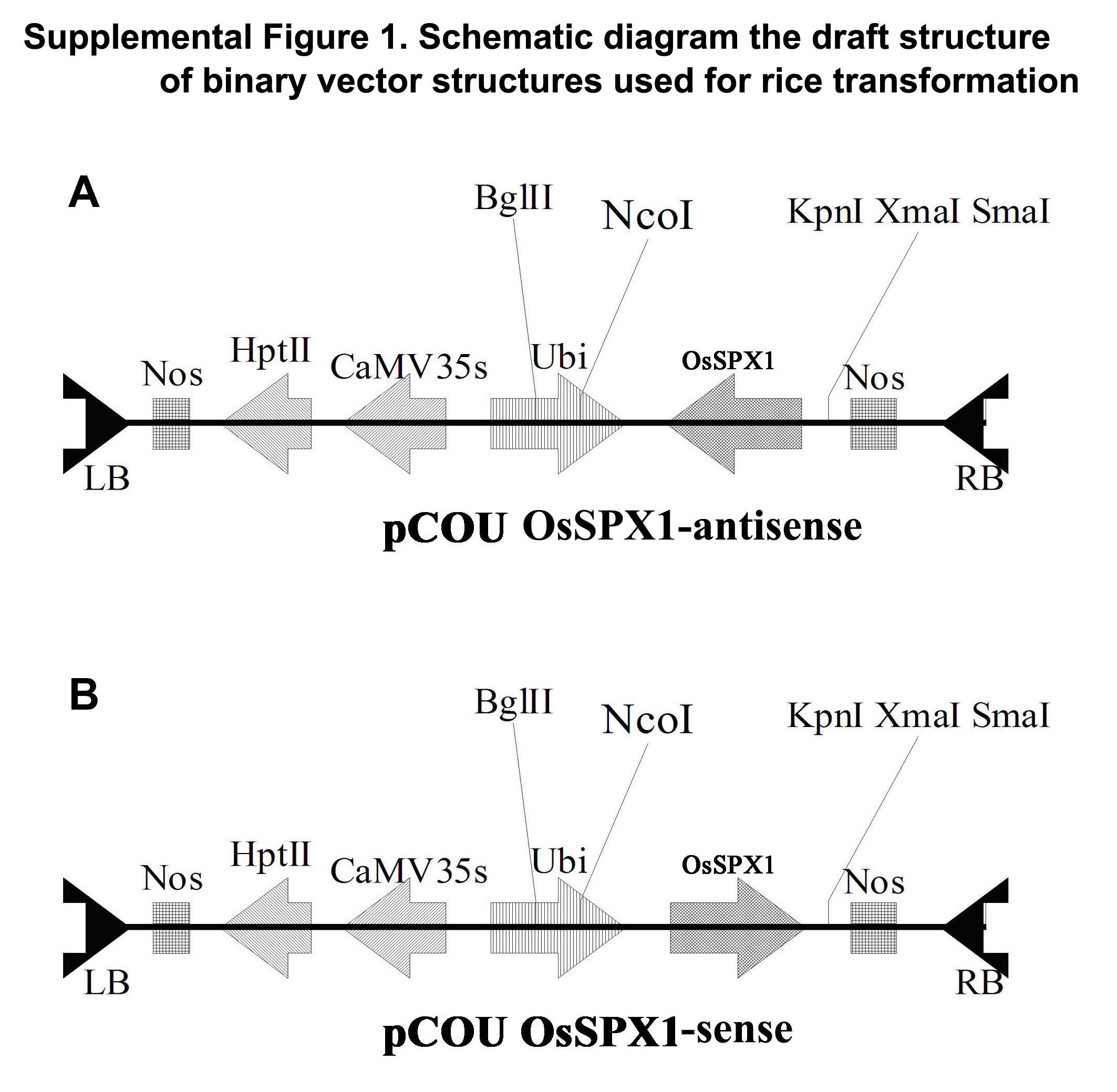

Supplement: Figure S1 — Schematic diagram of the draft structure of binary vector structures used for rice transformation. Construction scheme of plasmids with OsSPX1 in sense and antisense orientation. A: The binary vector pCOU OsSPX1-antisense was used for transgenic rice transformation. B: The binary vector pCOU OsSPX1-sense was used for transgenic rice transformation, which was adapted from our previous work [9]. LB and RB correspond to the T-DNA left and right borders. (JPG) [file pone.0081849.s001.jpg]
