# Supplementary material for: Down-Regulation of OsSPX1 Causes High Sensitivity to Cold and Oxidative Stresses in Rice Seedlings
Source: PLoS One. 2013 Dec 3;8(12):e81849. doi: 10.1371/journal.pone.0081849 (PMC3849359; doi:10.1371/journal.pone.0081849)
Supplement: Table S2 — Primer list of probe sets for real-time RT-PCR. (DOC) [file pone.0081849.s003.doc]

**Supplemental Table 2. Primer list of probe sets for real-time RT-PCR**

| **Unigene ID** | **Forward** | **Reverse** |
| --- | --- | --- |
| LOC_Os06g40120 (CDS region) | GTCGCGGCGCTTAGGTCTC | TGCCGGTCGTGTCATTTG |
| LOC_Os06g40120 (UTR region) | GTACACTGCATGACCTTGATCTTGA | CCGAGAGTTCATGAAAGAGGATGTAG |
| LOC_Os05g48390 (OsPHO2)^(a)^ | CGAGAATTTTGTCAAGGAGCA | TCACGAGCATGTCCAACAA |
| LOC_Os10g38600 | ACAGGGCAGTGGAGTACACC | GACGCCTGCTTAAGCTGAGT |
| LOC_Os10g38540 | TCTCATCGTCACTGCTTTGG | ATGACCCGGAAGAACATCAC |
| LOC_Os10g38350 | AAGAGCGATCTCCTCGTCAG | GTACTGCACGATGACCATGG |
| LOC_Os01g72150 | GTCATCGTCGAGTACGTCGA | ATGAATTGAGCCCAGAAACG |
| LOC_Os01g72140 | AGTGGACTGGCTCACTGGAT | GGCGTAGAGCAGGATACTCG |
| LOC_Os01g43774 | CCATGGTTGTTCTGAGCATG | GTTTGTCCCTCCCAAACAGA |
| LOC_Os07g23570 | CCTAAGGCTGTACAGCCCTG | ATCGGGATCGTCAATAGTGC |
| LOC_Os09g25070 | TGGTGCAAGTGCTCAAGAAC | CAGGAATGTGTGGGATTTGA |
| LOC_Os02g08440 | TCTTCTCTCCTTGGCTCAGC | ATCGGCGAACGATTTATCAC |
| LOC_Os06g37300 | TGCACTGCTAGGGGAGAAAT | AAACGTTGGCGTCGTACATT |
| LOC_Os11g47500 | AATTACTTGAGCCCGTGCAT | TGAGAATGGCCTACGTAGGG |

(a) The primer sequence from Zhou J. et al., 2008
